# Anesthetic Management in a Patient With Tethered Spinal Cord Syndrome Undergoing Knee Replacement Surgery

**DOI:** 10.1155/cria/2599778

**Published:** 2026-06-21

**Authors:** Lucas Alessi, Adrianne Bonham, Kiera Wendell, Jackson Eisenhauer, Saba Bolbolan, Elena Reshetnikoff, Richard Alessi, Matthew McEchron

**Affiliations:** ^1^ OMS IV College of Osteopathic Medicine, Rocky Vista University, Parker, Colorado, USA, rvu.edu; ^2^ Northern Colorado Anesthesia Professionals, Fort Collins, Colorado, USA; ^3^ Rocky Vista University College of Osteopathic Medicine, Parker, Colorado, USA

**Keywords:** anesthetic management, cerebrospinal fluid, conus medullaris, general anesthesia, lumbar spine MRI, spinal anesthesia, tethered cord syndrome, total knee replacement

## Abstract

Tethered spinal cord syndrome (TSCS) is a neurological disorder characterized by tissue attachments limiting spinal cord movement, which can result in the conus medullaris terminating at a level lower than the typical L1. This abnormality can cause significant complications during spinal anesthesia due to the risk of injury at the traditional puncture sites of L4‐L5. This case report discusses the anesthetic management of a 65‐year‐old female patient diagnosed with TSCS with her spinal cord extending to L5‐S1. She presented for a right total knee replacement. The patient had a history of successful spinal anesthesia for her previous left knee replacement but was apprehensive about repeating the procedure due to her spinal anatomy and the associated risks. A thorough preoperative assessment was conducted with this patient. This included a detailed MRI review by a radiologist and a neurosurgeon, which highlighted the risks associated with spinal anesthesia. Given the minimal cerebrospinal fluid around critical levels of the spinal cord, the risk of injury during spinal anesthesia was significant. After discussion with the patient about the risks and benefits of each anesthetic option, the care team and patient jointly decided that general anesthesia would offer the safest approach. General anesthesia was administered successfully with standard intraoperative monitoring and airway management. A preoperative adductor nerve block was also performed to manage postoperative knee pain. The patient emerged from anesthesia without complications, with stable vital signs and good neurovascular function. The interdisciplinary approach and careful preoperative planning were crucial to achieve a positive outcome in this case. This case highlights the importance of individualized anesthetic planning for patients with TSCS. It underscores the need for thorough preoperative assessment and patient‐centered care to determine the safest anesthetic approach. This report also reviews the mechanisms of spinal cord injury during neuraxial anesthesia in TSCS, discusses epidural anesthesia as an alternative, and synthesizes evidence‐based recommendations for preoperative assessment in the absence of formal guidelines. Further research and the development of formal guidelines for anesthetic management in TSCS are warranted to improve patient safety and outcomes.

## 1. Introduction

**FIGURE 1:**
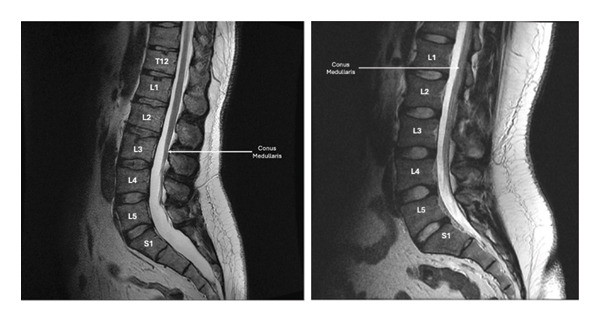
Left image: MRI showing the conus medullaris located abnormally at the L3 level in a 35‐year‐old male with tethered spinal cord syndrome (TSCS). Case courtesy of Krzysztof Nocoń, Radiopaedia.org, rID: 97956 [2]. Right Image: MRI demonstrating a normal conus medullaris position at the lower one‐third of the L1 vertebral body. Case courtesy of Bruno Di Muzio, Radiopaedia.org, rID: 43051 [3].

Tethered spinal cord syndrome (TSCS) is defined as a nervous system disorder in which tissue attaches to the spinal cord, limiting its movement [1]. The syndrome may be present from birth or occur during adulthood. TSCS may occur due to neural tube defects, or it may develop as the result of a spinal cord injury which can form scar tissue or a cyst [1]. A tethered spinal cord can cause the terminus of the conus medullaris to be more variable than those without. The normal terminus occurs around L1; however, in a patient with TSCS, the terminus may be as low as L5, as seen in Figure 1. The descent of the spinal cord can result in a number of problems in adults such as pain, sensory and motor problems, and loss of bowel and bladder control [1]. Due to the abnormal location of the terminus in TSCS, careful planning is required in surgical cases where spinal anesthesia may be an option.

Anesthesiologists should be cautious when performing spinal anesthesia in patients with TSCS. Typical needle puncture for spinal anesthesia occurs around the level of L2‐L3. In patients with TSCS, this can cause injury to the spinal cord because it extends beyond that level. The mechanism of injury during spinal anesthesia in TSCS patients is typically categorized into three pathways: direct trauma, local anesthetic neurotoxicity, and secondary cord ischemia [4, 5]. As the spinal needle approaches from the posterior direction, the dorsal columns of the cord are the first neural structures encountered. Thus, sensory deficits, including paresthesia, numbness, and neuropathic pain, represent the most common presentation following needle contact with a low‐lying conus [5]. However, if the needle penetrates deeper and anesthetic is deposited directly to the cord tissue, rather than the subarachnoid space, the result can be more severe—ranging from variable motor deficits to complete paralysis. Independent of trauma, disruption of spinal cord vasculature during needle insertion can produce secondary ischemia and epidural or subdural hematoma formation, which may cause additional mechanical compression [4]. Chronic longitudinal traction, as it occurs in a tethered cord, compromises baseline blood flow and oxidative metabolism within the parenchyma [6]. This means that even minor mechanical contact—which might only produce transient symptoms in a cord with normal perfusion—can result in more severe or permanent neurological injury in a tethered cord.

The literature describes several case studies of patients with TSCS who received spinal anesthesia which resulted in complications [7, 8]. One of the case studies describes a patient with TSCS in which her conus medullaris ended at L5. Spinal anesthesia was administered in the patient at the L4‐L5 level for an anti‐incontinence surgery. Following the surgery, the patient complained of pain along the L4‐L5 dermatomes but had normal motor function and reflexes [7]. A similar case involved a pregnant 25‐year‐old female patient with undiagnosed TSCS who received epidural anesthesia at the level of L2‐L3 [8]. This resulted in several complications following the procedure, including numbness in the extremities bilaterally, bilateral muscle weakness, and decreased reflexes below the knee joints [8].

A previous case series conducted by Liu et al. described complications in four patients with undiagnosed TSCS who received epidural or spinal anesthesia [4]. During needle insertion for spinal or epidural anesthesia, all patients reported radiating pain in the lower extremities and perineal region. These symptoms resolved following the anesthesia, and the anesthetic effects for all patients were successful. Several days following the surgeries, all patients reported weakness or paresthesia in the lower extremities. All patients later underwent magnetic resonance imaging which revealed TSCS with the end of the conus medullaris terminating between L3 and L5. Interestingly, prior to the surgery, all patients in this study reported occasional pain in the back, lower extremities, and perineal region. This case series along with similar case reports [9] highlights the importance of performing an extensive preoperative history. Moreover, the symptoms revealed in the preoperative history could lead clinicians to suspect possible TSCS and order further imaging.

### 1.1. Anesthetic Alternatives and Considerations in TSCS

Spinal anesthesia is commonly used for lower extremity surgeries such as total knee replacement, as well as surgeries involving the inguinal region [10]. A recent study compared general anesthesia and spinal anesthesia use with total knee replacement. The study showed that spinal anesthesia patients had lower rates of postoperative adverse events, including blood transfusion, deep vein thrombosis, and readmission [10]. However, it also showed that there were minimal differences between these groups when examining the early patient outcomes after the total knee replacement [10]. Thus, spinal anesthesia may have certain advantages compared to general anesthesia, especially for total knee replacement; however, general anesthesia is still a safe method if spinal anesthesia is unavailable.

Despite the advantages of spinal anesthesia for certain surgical procedures, it has been classified as an absolute contraindication in patients with a posteriorly tethered cord [11]. Given the risks of spinal anesthesia in TSCS, epidural anesthesia has been proposed as a safer neuraxial alternative. The distinction between the two is that an epidural remains in the extradural space and does not puncture the dura mater; therefore, it does not enter the subarachnoid space where a low‐lying conus is at risk for direct contact [11]. However, epidurals are not without risk and limitations in TSCS patients. Fibrotic tissue and adhesions associated with the tethering of the cord can impair the spread of anesthetic within the epidural space which may result in an inadequate or patchy block. Scher et al. reported a case in which both epidural and spinal anesthesia failed in a patient with an unrecognized tethered cord, necessitating conversion to general anesthesia, highlighting the structural abnormalities that prevented adequate anesthetic effect [12]. The risk of accidental dural puncture must also be considered as this would recreate the same hazard as spinal anesthesia.

### 1.2. Patient Information

A 65‐year‐old female with a history of hypertension, obesity, type 2 diabetes mellitus, and hyperlipidemia presented for a right total knee replacement. She had no history of heart attacks or strokes and was a nonsmoker. She had previously undergone a left knee replacement with uneventful spinal anesthesia 1 year prior and was apprehensive about undergoing another spinal anesthesia due to her known tethered spinal cord, diagnosed when she was a teenager. Her spinal anesthesia may have been successful despite her tethered spinal cord due to multiple factors. First, slight variations in needle angle or depth at the same interspace can determine whether the needle contacts cord tissue or passes through the subarachnoid space without incident [5]. Second, patient positioning during the prior surgery may have allowed redistribution of cerebrospinal fluid, providing a temporary buffer around the cord that is likely not reproducible. Third, interval degenerative changes that may have occurred in the year between surgeries—including disc space narrowing, facet hypertrophy, or ligamentum flavum thickening—can further reduce the minimal cerebrospinal fluid space around the cord, making a repeat procedure more dangerous than the first, particularly given her age, obesity, and comorbid conditions. In order to evaluate the current validity of performing a repeat spinal anesthesia, an MRI was ordered and confirmed the conus medullaris extending to L5‐S1 with minimal cerebrospinal fluid around the cord from L1–L5. Given her apprehension and anxiety surrounding possible complications due to her spinal anatomy, the care team knew that a discussion covering the potential risks and benefits of the various anesthetic approaches would be necessary.

### 1.3. Clinical Assessment and Challenges

In preparation for the procedure, a thorough preoperative assessment was conducted. This included a detailed review of her MRI by both a radiologist and a neurosurgeon. The main clinical challenge was managing her tethered cord with the conus medullaris extending to L5‐S1 and the minimal cerebrospinal fluid around critical levels, which significantly increased the risk of complications from spinal anesthesia. Given the limited cerebrospinal fluid, there was a significant risk that the needle would be inserted into the spinal cord without ever seeing any spinal fluid, thus potentially causing permanent damage. A thorough discussion was held with the patient about the risks and benefits of spinal versus general anesthesia, considering her specific condition. Spinal anesthesia is generally preferred for lower extremity joint replacements due to several benefits, including less blood loss, fewer blood clots, less stress on the cardiopulmonary system, less risk of exacerbating memory issues, lower risk of aspiration, and decreased risk to dental structures from intubation [10]. However, in this case, the risks of spinal anesthesia outweighed these benefits due to the patient’s tethered cord condition. Ultimately, it was decided that general anesthesia would be a safer option with this patient.

### 1.4. Management and Intraoperative Course

The patient consented to general anesthesia after understanding the potential risks and benefits, which alleviated her apprehension. Standard intraoperative monitoring was employed, including ECG, blood pressure, and oxygen saturation. General anesthesia was induced using standard agents, including propofol, fentanyl, lidocaine, rocuronium, and sevoflurane, with airway management successfully secured via endotracheal intubation. A preoperative adductor nerve block was performed to help with anterior knee pain postoperatively. Anesthesia was maintained with inhalational agents and IV medications, with continuous monitoring of the depth of anesthesia and physiological parameters.

### 1.5. Emergence and Postoperative Care

The patient emerged from anesthesia uneventfully, with stable vital signs in the recovery room and good neurovascular function. The perioperative course was smooth, and the patient was reassured and satisfied with the care she received. The successful management of her anesthesia without complications highlighted the effectiveness of the interdisciplinary approach and thorough preoperative planning.

## 2. Discussion

The management of anesthesia in patients with TSCS requires careful preoperative planning due to the challenges posed by the condition. The literature highlights complications associated with spinal and epidural anesthesia in TSCS patients, often resulting from the abnormal positions of the conus and reduced cerebrospinal fluid. Case studies of TSCS have documented nerve injuries, paresthesia, and muscle weakness following needle punctures at levels presumed to be safe [7, 8]. These findings emphasize the importance of thorough preoperative assessments, including imaging, to understand the patient’s spinal anatomy and mitigate potential risks. In this case, collaboration among radiologists, neurosurgeons, and anesthesiologists allowed the care team to navigate these challenges effectively and develop a safe anesthetic plan.

Regarding the safety of spinal anesthesia in TSCS, the literature does not categorically prohibit it in all cases. Liu et al. concluded that spinal anesthesia should be prohibited in adult TSCS patients; however, all four patients in their series had undiagnosed tethered cords [4]. This distinction is clinically important: the danger is not only the tethered cord itself but rather performing neuraxial anesthesia without knowledge of where the cord terminates. If preoperative MRI confirms that the conus medullaris terminates at a level—such as L3—that leaves adequate subarachnoid space at the intended puncture site (e.g., L4‐L5), spinal anesthesia could theoretically be performed safely, as the needle would enter a region containing only cauda equina nerve roots. Prerequisites for such an approach would include the following: (1) recent MRI confirming the exact level of conus termination and adequate cerebrospinal fluid volume at the intended puncture site; (2) preprocedural bedside ultrasound to confirm the correct interspace, as landmark‐based identification is unreliable in patients with spinal abnormalities; and (3) an experienced operator. In cases such as the patient presented here, where the conus extends to L5‐S1 and there is minimal cerebrospinal fluid throughout the lumbar spine, there is essentially no safe access point, and general anesthesia is the clear choice.

The decision to use general anesthesia in this patient highlights the lack of clear guidelines for managing anesthesia in TSCS cases. While spinal anesthesia is often preferred for lower extremity surgeries due to its benefits, including reduced blood loss and postoperative complications, it was not the safest option in this scenario. Although standardized protocols have many benefits, this case underscores the need for weighing the risks and benefits of all options by incorporating advanced imaging, interdisciplinary consultation, and patient input to guide decision‐making—a particularly relevant consideration given the current absence of formal guidelines for anesthetic management in TSCS.

Several evidence‐based recommendations can be synthesized from existing literature to guide clinical decision‐making. The American Society of Regional Anesthesia Practice Advisory on spinal cord injury associated with regional anesthesia has emphasized the importance of understanding patient‐specific spinal anatomy and avoiding needle insertion at vertebral levels where the spinal cord may be present [5]. For patients with known or suspected TSCS, preoperative MRI should identify four key anatomical features: (1) the level of conus termination, (2) the presence and location of tethering, (3) the volume of the cerebrospinal fluid at the intended puncture level, and (4) any intraspinal masses [11]. Repeat diagnostic confirmation is not needed with repeat imaging for each subsequent surgical encounter; however, if the most recent MRI is several years old, interval degenerative changes—including progressive stenosis and reduced cerebrospinal fluid volume—may have altered the risk profile at the intended puncture level, and updated imaging for procedural planning purposes may be warranted. Regarding consultation, an interdisciplinary approach involving radiology, neurosurgery, and anesthesia colleagues should be considered standard practice when neuraxial anesthesia is being contemplated in a TSCS patient [12]. Clinical signs that should prompt further investigation prior to neuraxial anesthesia include cutaneous stigmata over the lumbosacral spine (hairy nevus, sacral dimple, and hemangioma), foot deformities, chronic back or lower extremity pain, perineal pain, and bladder or bowel dysfunction, as they may be signs of undiagnosed TSCS [13].

Several key lessons can be taken from this case for clinicians managing patients with known or suspected spinal cord abnormalities. Most importantly, patient apprehension should be treated as a clinical sign rather than dismissed as anxiety. The patient’s concern about repeating spinal anesthesia reflected a legitimate awareness of her own anatomy. This led to a subsequent MRI review and neurosurgical consultation revealing anatomy that fundamentally changed the anesthetic plan. Had the team defaulted to the prior uneventful spinal without further workup, the outcomes could have been catastrophic [12]. This case also demonstrated the value of shared decision‐making—the patient participated in choosing general anesthesia, which not only improved safety but also reduced her anxiety and contributed to her overall satisfaction with care. Finally, clinicians should maintain a low threshold for additional workup when patients with complex medical histories raise concerns about planned procedures [4].

## 3. Conclusion

This case underscores the importance of individualized anesthetic planning, thorough preoperative assessment, and the unique challenges posed by rare conditions like TSCS, for which no clear anesthetic guidelines currently exist. Moreover, general anesthesia can be a safer option for patients with complex spinal anatomy undergoing lower extremity surgery, despite the usual benefits of spinal anesthesia for such procedures. A patient‐centered approach and effective communication were crucial for achieving a positive outcome in this case. Further research and the development of formal guidelines for anesthetic management in TSCS are warranted to standardize preoperative assessment and improve patient safety. This case also highlights the importance of quality imaging of the spinal cord prior to anesthesia if there is a complex medical history.

## Funding

No funding was received for this manuscript.

## Conflicts of Interest

The authors declare no conflicts of interest.

## Data Availability

Data sharing is not applicable to this article as no datasets were generated or analyzed during the current study.
